# Spatiotemporal distribution of *Ceratonova shasta* in the lower Columbia River Basin and effects of exposure on survival of juvenile chum salmon *Oncorhynchus keta*

**DOI:** 10.1371/journal.pone.0273438

**Published:** 2022-08-26

**Authors:** Kristen Homel, Julie D. Alexander

**Affiliations:** 1 Oregon Department of Fish and Wildlife, Corvallis, Oregon, United States of America; 2 Department of Microbiology, Oregon State University, Corvallis, Oregon, United States of America; University of Idaho, UNITED STATES

## Abstract

In the Columbia River Basin (CRB), USA, anthropogenic factors ranging from dam construction to land use changes have modified riverine flow and temperature regimes and degraded salmon habitat. These factors are directly implicated in native salmon and steelhead (*Oncorhynchus* species) population declines and also indirectly cause mortality by altering outcomes of ecological interactions. For example, attenuated flows and warmer water temperatures drive increased parasite densities and in turn, overwhelm salmonid resistance thresholds, resulting in high disease and mortality. Outcomes of interactions between the freshwater myxozoan parasite, *Ceratonova shasta*, and its salmonid hosts (e.g., coho *O*. *kisutch* and Chinook salmon *O*. *tshawytscha*) are well-described, but less is known about effects on chum salmon *O*. *keta*, which have a comparatively brief freshwater residency. The goal of this study was to describe the distribution of *C*. *shasta* relative to chum salmon habitat in the CRB and assess its potential to cause mortality in juvenile chum salmon (listed as threatened in the CRB under the U.S. Endangered Species Act). We measured *C*. *shasta* densities in water samples collected from chum salmon habitat throughout the lower CRB during the period of juvenile chum salmon outmigration, 2018–2020. In 2019, we exposed caged chum salmon fry from two hatchery stocks at three *C*. *shasta*-positive sites to assess infection prevalence and survival. Results demonstrated: (1) *C*. *shasta* was detected in spawning streams from which chum salmon have been extirpated but was not detected in contemporary spawning habitat while juvenile chum salmon were present, (2) spatiotemporal overlap occurs between *C*. *shasta* and juvenile chum salmon in the Columbia River mainstem, and (3) low densities of *C*. *shasta* caused lethal infection in chum salmon fry from both hatchery stocks. Collectively, our results suggest *C*. *shasta* may limit recovery of chum salmon now and in the future.

## Introduction

In the Columbia River Basin (CRB), USA, anthropogenic impacts, such as construction of large dams and changes in land use, have altered flow and temperature regimes and reshaped freshwater habitat over the last century [1]. These impacts have been directly associated with population declines of native salmon and steelhead (*Oncorhynchus* species) [2,3], resulting in significant effort directed towards addressing habitat degradation and decreasing mortality during passage through dams [e.g., 4]. Comparably less effort has focused on understanding the indirect impacts of habitat degradation, which may also be a significant source of mortality [e.g., 5,6]. For example, habitat degradation and attenuated flows may produce conditions that allow endemic parasites to rapidly increase in density [7,8], upsetting host-parasite ecological interactions. Although salmonids that have co-evolved with parasites maintain natural resistance to disease, high parasite densities can exceed the salmonid host’s threshold of resistance and result in high disease-related mortality [9–11].

The myxozoan parasite *Ceratonova shasta* [12] causes infection and mortality in salmonids and is endemic in river systems throughout the Pacific Northwest [10,11,13,14]. *Ceratonova shasta* alternates between infecting a salmonid and an invertebrate host, and two waterborne spore stages during its life cycle [14]. The invertebrate host (*Manayunkia occidentalis*) ingests myxospores and releases actinospores into the water column [14,15]. Actinospores infect fish hosts via the gills (most typical), and *C*. *shasta* migrates through the circulatory system before developing into myxospores in the intestinal tissues [16]. In the fish host, parasite proliferation can cause severe intestinal hemorrhaging and death [17,18]. The *C*. *shasta* life cycle is temperature dependent and both adult and juvenile salmonid stages can become infected and diseased [9].

Specific effects of *C*. *shasta* vary among salmonid species and geographic locations. Different strains of *C*. *shasta* (genotypes 0, I, and II; [19,20]) vary in specificity for, and virulence in, their respective hosts [21]; genotype 0 infects but does not typically cause disease in steelhead *O*. *mykiss*, genotype I causes disease in Chinook salmon *O*. *tshawytscha*, and genotype II is a generalist, causing disease in coho *O*. *kisutch*, chum *O*. *keta*, and sockeye salmon *O*. *nerka*, and allopatric rainbow trout *O*. *mykiss* [19–21]. Sympatric evolution of the host and *C*. *shasta* produces resistance to disease [9,10,22], but resistance can be overwhelmed at high parasite densities. For example, Klamath River salmonids exhibit resistance to infection and disease but in some years high densities of *C*. *shasta* (>10 spores/L) have been associated with population level impacts due to significant (>90% infection and >70% mortality) juvenile salmon mortality [23–26]. Salmonids native to the CRB would have evolved similar resistance to *C*. *shasta*, but epizootics have occurred in hatchery coho and Chinook salmon at increased parasite densities [27,28]. Likewise, both infection and disease have been reported in juvenile Chinook salmon, coho salmon, and steelhead captured during their migration down the Columbia River [7].

Previous work in the CRB, suggests the *C*. *shasta* stage that is infectious for salmoninds (actinospores) is present seasonally and distributed broadly. Sentinel exposures (caged salmon held *in situ*) demonstrate actinospores are present beginning in late spring, coinciding with increasing water temperatures (>10°C; [14]), through early fall [7,17,29]. Results from sentinel exposures also demonstrate the actinospore stage is distributed in the Columbia River mainstem [30] from the mouth upstream to confluence with the Snake River [31]. The parasite has also been detected in several major tributaries to the Columbia River including the Deschutes and the Willamette Rivers [17,22,31]. Research on *C*. *shasta* has largely focused on salmonid species that exhibit extended juvenile freshwater rearing due to the potential for prolonged overlap between the juvenile salmonids and the *C*. *shasta* actinospore stage. Much less is known about the effects of *C*. *shasta* exposure on species exhibiting a brief freshwater residency, such as chum salmon. In the CRB, chum salmon are listed as threatened under the U.S. Endangered Species Act [32] and understanding the potential risks presented by *C*. *shasta* is critical for management and recovery efforts.

Columbia River chum salmon were historically distributed in the Columbia River upstream to Celilo Falls (river kilometer [rkm] 320) and spawned in both mainstem and tributary habitats. An estimated 1,000,000 adults returned to spawn in 1928, [33,34], but abundance declined to < 1,000 adults over a period of several decades [35,36], prompting ESA-listing in 1999 [32]. Of 17 historical chum salmon populations, only the Grays River and Lower Gorge populations are considered viable. Columbia River chum salmon exhibit an ocean-type life history [37]; spawning peaks in late November and fry outmigrate the following spring [34]. Fry inhabit the estuary anywhere from days [38] to months [39,40] before entering the ocean by June [41]. Prior to entering brackish habitat in the estuary, *C*. *shasta* actinospores may co-occur with juvenile chum salmon during the later portion of fry residency in natal tributaries and while rearing in the freshwater portion of the lower Columbia River. *C*. *shasta* infections and mortality have been reported in juvenile chum salmon where they co-occur in British Columbia, Alaska, and Coastal Oregon [13,42–44].

The goal of this study was to investigate spatial overlap between *C*. *shasta* and Columbia River chum salmon habitat and to assess the potential for *C*. *shasta*-related mortality. Our specific objectives were to (1) describe the spatiotemporal distribution and density of *C*. *shasta* genotypes relative to the contemporary and historical distributions of chum salmon, and (2) describe the susceptibility of juvenile chum salmon from the Lower Gorge and Grays River populations to infection from exposure to *C*. *shasta* in the Columbia River and tributaries. To determine *C*. *shasta* distribution and density, we quantified *C*. *shasta* densities in water samples from Columbia River mainstem and tributary sites during the period (Feb–May) when juvenile chum salmon would co-occur, 2018–2020. To assess susceptibility of juvenile chum salmon to *C*. *shasta*, we held fry from Lower Gorge and Grays River-origin hatchery stocks in sentinel cages at three sites in 2019 where *C*. *shasta* was previously detected (2018), measured *C*. *shasta* densities during exposure, and subsequently assessed mortality and myxospore production in infected fish. We interpret our results in the context of potential risks *C*. *shasta* poses to the recovery of Columbia River chum salmon.

## Materials and methods

### Study area

**Fig 1:**
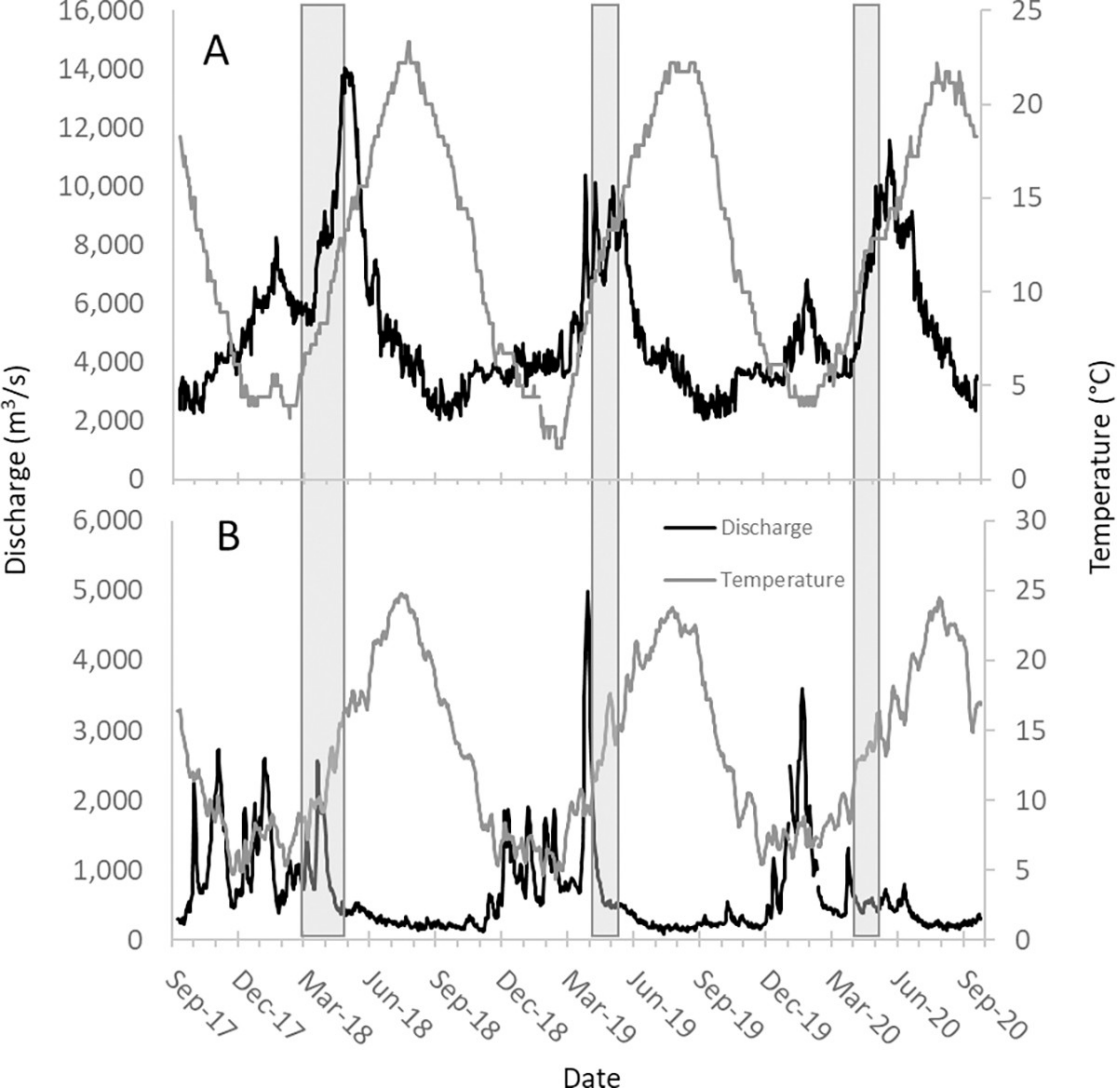
**Water temperature (°C) and discharge (m**^**3**^**/s) in the Columbia River at Bonneville Dam (panel A; USGS gage site 453845121562000) and the Willamette River at Portland, Oregon (panel B; USGS gage site 14211720) 2018–2020.** Grey bars denote the timeframe water sampling occurred each year. Note different in Y-axis scale between panels.

The CRB drains an area of 668,000 km^2^. This study was conducted in the lower portion of the basin from Bonneville Dam (rkm 234) downstream to the estuary. This section of river is tidally influenced, although the upstream extent of salt water is typically constrained by Columbia River discharge to approximately rkm 25 [45]. The flow regime of the Columbia River is regulated by large main stem dams, which results in earlier and attenuated peak flows, relative to the historical unregulated hydrograph [46]. In the lower Columbia River, the hydrographs of tributaries draining off the Cascade Range are influenced by both fall and winter rain events and snowmelt into early summer. The hydrographs of tributaries draining the Columbia River Gorge and Coast Range are primarily rain dominated. Average daily water temperatures in the lower Columbia River approach 4°C in the winter and can exceed 21°C in the summer (available from: Fish Passage Center; https://www.fpc.org/WebForm2013/NETHistoric_tempgraph.aspx). To represent the range of environmental conditions present during field sampling, discharge (m^3^/s) and temperature (°C) data were compiled from the USGS gages located on Columbia River at Bonneville Dam and the Willamette River at Portland, Oregon (USGS gage numbers 453845121562000 and the 14211720, respectively; Fig 1).

### Objective 1: Spatiotemporal distribution of *C*. *shasta*

The distribution of *C*. *shasta* was described using water samples collected throughout the lower Columbia River and tributaries from 2018 through 2020. Sampling was stratified to target sites where chum salmon (1) currently spawn (*n* = 11; hereafter termed “contemporary” spawning streams), (2) historically spawned but are now extirpated (*n* = 5; hereafter termed “historical” spawning streams”) or (3) intermittently spawn (*n* = 13; hereafter termed “intermittent spawning streams”), in addition to sites on mainstem Columbia River where juvenile chum salmon rear and migrate before entering the ocean (*n* = 29; hereafter “mainstem rearing habitat”). All sites were sampled either once annually to characterize parasite distribution (spatial sampling; large number of sites) or multiple times annually to characterize temporal variation in parasite abundance (temporal sampling; fewer sites but more sampling events). Permits were not required to access sample sites. Columbia River samples were all collected at public access sites. Most tributary samples were also collected at public access sites, and samples collected on private property were done so with the explicit permission of the landowner.

Sample dates were selected to overlap with the period when chum salmon fry were present and also to extend briefly past that date to capture any interannual variation in *C*. *shasta* presence or juvenile migration timing. This variation can be observed in small populations with variable spawn timing [47]. In general, chum salmon fry migrate from tributaries in Oregon beginning at the end of February and extending as late as mid-May [47]. In Washington, fry migrate at the beginning of February and have left their spawning tributaries by mid-April [48]. Juveniles inhabit the Columbia River primarily March–May [41].

**Fig 2:**
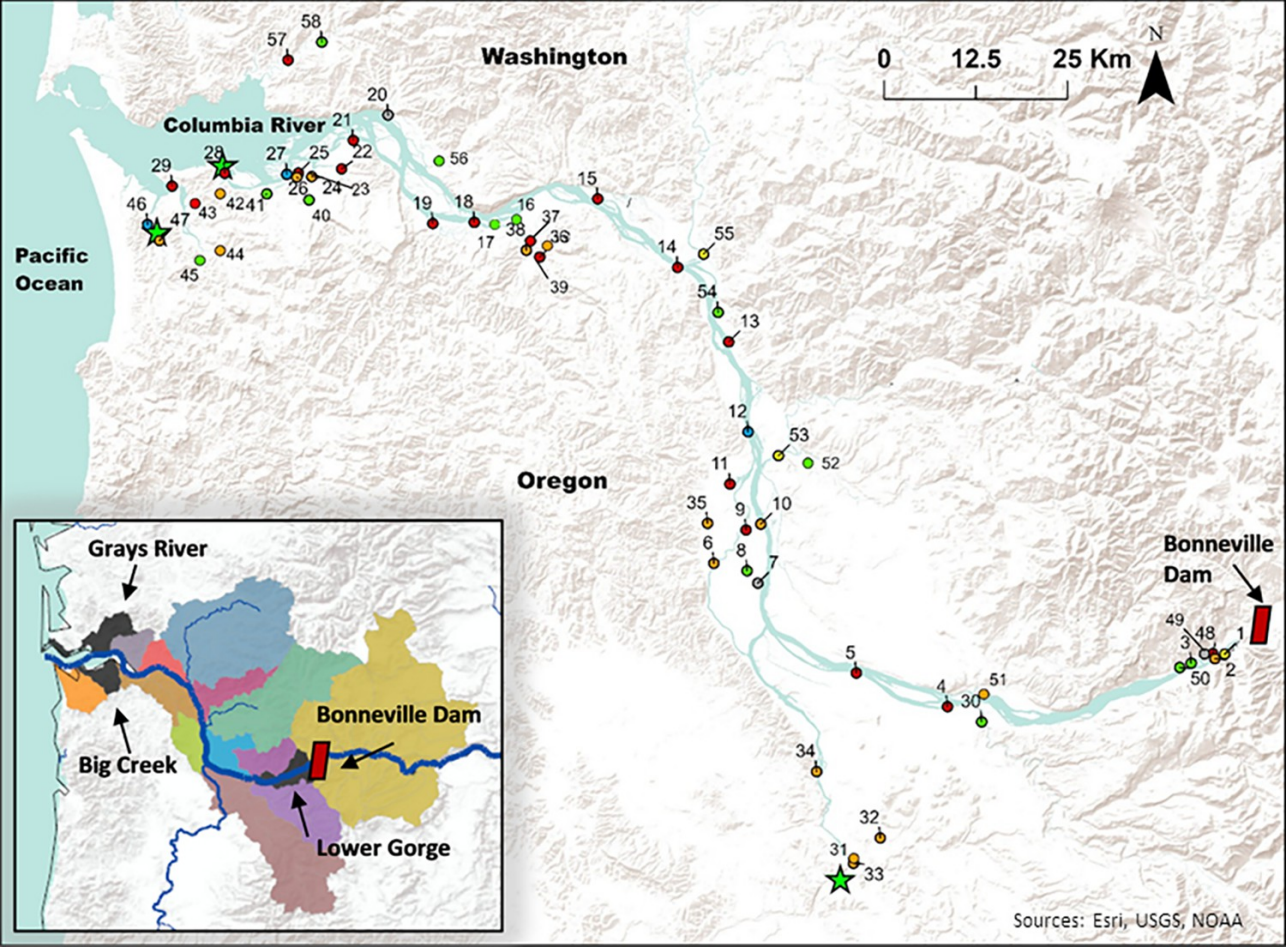
Water sample locations for *Ceratonova shasta* in the Columbia River Basin, March 15 –May 1, 2018, April 15 –May 15, 2019, and April 15 –May 15, 2020. Markers are labeled with the site code in Tables 1 and 2. Marker color indicates the genotypes of *C*. *shasta* that were detected (green = no C. shasta, yellow = genotype I, red = genotype II, orange = genotypes I and II, grey = *C*. *gasterostea*, and blue = sample could not be genotyped). Green stars indicate sites where sentinel cages were held (Lewis and Clark at site 46, Tongue Point at site 28, and Willamette at site 33). Inset shows boundaries for 17 historical populations; chum salmon *Oncorhynchus keta* were collected for the sentinel study from the Grays River, Big Creek, and Lower Gorge Populations (shown in black). Topographic basemap was created by Esri, USGS, and NOAA.

A core set of spatial and temporal sites was sampled annually from 2018 through 2020. In 2019 and 2020, additional sites were included to better characterize distribution, and sampling dates were modified to focus on the time frame in 2018 when *C*. *shasta* was detected throughout the study area. In 2018, temporal sites (*n* = 17) were sampled biweekly March 1 –May 1, and spatial sties (*n* = 18) were sampled on May 1 (Figs 1 and 2; Tables 1 and 2). In 2019, temporal sites (*n* = 19) were sampled biweekly April 15 –May 15 and spatial sites (*n* = 22) were sampled on May 1 (Figs 1 and 2; Tables 1 and 2). In 2020, some sites could not be accessed due to the coronavirus pandemic. Spatial sampling occurred on May 7 for Columbia River sites (*n* = 12) and May 15 for tributary sites (*n* = 10). Temporal sampling (*n* = 15) occurred weekly from April 15 –May 15 (Figs 1 and 2; Tables 1 and 2), except when affected by temporary closures. The study area map was created using ArcGIS ® Pro (version 3.0) and is the intellectual property of Esri and used herein under license. Copyright © Esri. All rights reserved. The topographic basemap was created by Esri, USGS, and NOAA.

**Table 1:** Site code, site name, and sample type by year for Columbia River sites sampled for *Ceratonova shasta*, 2018–2020. “X” indicates site not sampled during a given year.

Site code (State)	Site name	2018	2019	2020
1 (OR)	Bonneville Hatchery	Temporal	Temporal	Spatial
2 (WA)	North Bonneville	Temporal	Temporal	Spatial
3 (WA)	Beacon Rock	Spatial	Spatial	X
4 (OR)	Chinook Landing	X	X	Temporal
5 (OR)	Broughton Beach	X	X	Spatial
6 (OR)	Multnomah Channel	Spatial	Spatial	Temporal
7 (OR)	Dairy Creek	X	X	Spatial
8 (OR)	Sturgeon Lake	X	X	Spatial
9 (OR)	Gilbert River	X	X	Spatial
10 (OR)	Sauvie Island lighthouse	X	X	Spatial
11 (OR)	Scappoose Bay Marina	X	X	Temporal
12 (OR)	Pixie Park at Columbia City	X	X	Spatial
13 (WA)	Upstream of Kalama River	X	X	Spatial
14 (OR)	Rainier City Park at Rainier	X	X	Spatial
15 (WA)	County Line Park near Longview	X	X	Spatial
16 (OR)	Wallace Island (1)	X	Spatial	X
17 (OR)	Wallace Island (2)	X	Spatial	X
18 (OR)	Jones Beach	X	X	Temporal
19 (OR)	Westport Slough	Spatial	Spatial	Temporal
20 (WA)	Downstream Skamokawa	X	X	Spatial
21 (OR)	Aldrich Point	X	X	Spatial
22 (OR)	Blind Slough	X	Spatial	X
23 (OR)	Knappa Slough (Dock)	Temporal	Temporal	Temporal
24 (OR)	Knappa Slough (main channel)	Spatial	Spatial	X
25 (OR)	Karlson Island	Spatial	Spatial	X
26 (OR)	Minaker Island (South)	Spatial	Spatial	X
27 (OR)	Minaker Island (West)	Spatial	Spatial	X
28 (OR)	Tongue Point (Sentinel)	X	Spatial	Spatial
29 (OR)	Youngs Bay	X	Spatial	Temporal

**Table 2:** Site code, site name, status of chum salmon *Oncorhynchus keta* spawning (historically present; intermittently present, currently present), and sample type by year for tributary sites sampled for *Ceratonova shasta*, 2018–2020 in the lower Columbia River Basin. “X” indicates site not sampled during a given year.

Site code (State)	Site name	Chum status	2018	2019	2020
30 (OR)	Sandy River	Historical	Temporal	Temporal	X
31 (OR)	Clackamas River (Clackamette Park)	Intermittent	Temporal	Temporal	X
32 (OR)	Clackamas River (Upstream)	Intermittent	X	X	Temporal
33 (OR)	Willamette River (Sentinel)	Intermittent	Temporal	Temporal	Temporal
34 (OR)	Willamette River (Willamette Park)	Intermittent	X	X	Temporal
35 (OR)	Scappoose Creek	Historical	Spatial	Spatial	X
36 (OR)	Stewart Creek	Historical	Temporal	Temporal	X
37 (OR)	Beaver Slough	Historical	Temporal	Temporal	Temporal
38 (OR)	Clatskanie River (lower)	Intermittent	Temporal	Temporal	Temporal
39 (OR)	Clatskanie River (upper)	Intermittent	Spatial	Spatial	Spatial
40 (OR)	Big Creek	Present	Temporal	Temporal	Spatial
41 (OR)	Bear Creek	Present	Temporal	Temporal	X
42 (OR)	Mill Creek	Intermittent	X	Spatial	X
43 (OR)	Wallooskee River	Intermittent	Spatial	Spatial	X
44 (OR)	Klaskanine River	Intermittent	Spatial	Spatial	X
45 (OR)	Youngs River	Historical	Spatial	X	X
46 (OR)	Lewis and Clark River (lower)	Intermittent	Temporal	Temporal	X
47 (OR)	Lewis and Clark River (upper; Sentinel)	Intermittent	Temporal	Temporal	Temporal
48 (WA)	Hamilton Creek	Present	Temporal	Temporal	Spatial
49 (WA)	Hardy Creek	Present	X	Spatial	Spatial
50 (WA)	Duncan Creek	Present	X	Spatial	Spatial
51 (WA)	Washougal River	Intermittent	Temporal	Temporal	X
52 (WA)	E Fork Lewis River	Present	Spatial	Spatial	X
53 (WA)	Lewis River	Present	Temporal	Temporal	Spatial
54 (WA)	Kalama River	Intermittent	Spatial	Spatial	Spatial
55 (WA)	Cowlitz River	Present	Temporal	Temporal	Spatial
56 (WA)	Elochoman River	Present	Spatial	Spatial	X
57 (WA)	Grays River (lower)	Present	Temporal	Temporal	Temporal
58 (WA)	Grays River (upper)	Present	X	Temporal	Temporal

Samples were collected and processed following the protocol of [49] with modifications as in [11]. At each site, the sample bottles were rinsed with river water, recapped, and positioned 10–30 cm below the water surface (depending on water depth with the aim of avoiding benthic disturbance). The cap was removed from the bottle once it was underwater to avoid collecting surface debris in the sample (4L). Samples were stored in a cooler on ice until they could be filtered within 24 hours of collection. Water samples were filtered using a vacuum filtration set up with a MF-Millipore filter membrane (nitrocellulose 5 μm pore size; [49]). In 2018, the entire (4L) sample was combined and filtered together, but in 2019 and 2020, liters were filtered separately to address the high inhibition observed in 2018 samples. Filters were stored in 2 ml centrifuge tubes at -20° C.

Total genomic DNA was extracted from filters according to the protocol described in [49]. We addressed inhibition using an internal positive control run with the *C*. *shasta* assay. Thus, inhibition was assessed for every single sample assayed. If inhibition was detected, the sample was diluted and re-run (dilution approaches included 1:4, 1: 10, or 1:100 and spore standards were also diluted to the relevant concentration). After dilution, sample C. *shasta* quantities were adjusted accordingly. Filter volume was consistent among years and all results were reported in spores/ L. In 2019 and 2020, variation in spore density among filters from a single site and date was assessed by calculating the coefficient of variation (CV) for each sample and then calculating an average CV across sites for the year.

The presence and density (spores/ L) of *C*. *shasta* was determined by qPCR (as in [11]). The qPCR assay reliably quantified spores only at densities ≥ 2 spores/ L [50]. Therefore, any positive detections below this threshold were reported as < 2 spores/ L, and quantities were reported only for samples measured above that threshold. Water samples positive for *C*. *shasta* by qPCR were sequenced to confirm the presence of *C*. *shasta* (the *C*. *shasta* assay also amplifies *C*. *gasterostea*, which infects coastal Sticklelback *Gasterosteus* sp.) and to determine genotype (0, I, and II; [19,20]). The proportion of each genotype was apportioned to the total spore quantity. Samples that were positive only for *C*. *gasterostea* were recorded as negative for *C*. *shasta*.

#### Analysis

We hypothesized that if *C*. *shasta* impacts juvenile chum salmon while they inhabit natal tributaries, we would observe a negative relationship between the distribution of *C*. *shasta* and the contemporary spawning distribution of chum salmon. Contemporary spawning distribution data were assembled from reports and spawn survey data (WDFW abundance data available at https://fortress.wa.gov/dfw/score/score/maps; [51]). Chum salmon were considered present if adults (“spawners”) were observed in the stream annually, even if at a low abundance. If spawners were not observed or only observed intermittently (potential strays), they were considered absent. A stream was considered positive for *C*. *shasta* if genotype II, which has been shown to infect chum salmon [52], was detected during sampling up to and including the spatial sampling on May 1; CRB chum salmon are not thought to be susceptible to infection by genotypes 0 and I. The May 1 date was selected as a conservative point to differentiate between a time period when juveniles were generally known to be present in spawning streams and could be exposed to *C*. *shasta* and a time period when they were generally absent (although a small number of wild chum salmon may be present in Oregon tributaries after this date). Positive detections after the May 1 cutoff date were reported but not included in the analysis. The contemporary distribution of chum salmon was compared to the contemporary distribution of *C*. *shasta* using a chi-squared analysis in Program R [53].

### Objective 2: Sentinel study

We measured prevalence of *C*. *shasta* infection and associated mortality in juvenile chum salmon exposed in sentinel cages to river water, May 1 –May 8, 2019. These dates were selected to ensure the sites would be positive for *C*. *shasta* during the sentinel exposure. Three sites were selected for the exposures to target the range of *C*. *shasta* densities (of genotype II) measured in water samples collected the previous sampling season (in 2018; Willamette River = high density, Lewis and Clark River = medium density, and Tongue Point = low density; Fig 2). Chum salmon fry were collected from two hatchery stocks: Big Creek (derived from adults collected in the Grays River population; weight = 1–1.5g), and the Washougal River (derived from adults collected in the Lower Gorge population; weight = 1–2.6 g). Fry were loaded into oxygenated coolers filled with water from their respective hatcheries and transported to the sentinel cage sites. Each hatchery stock was held in a separate cage at each sentinel site (*n* = 30 fish/ cage; *n* = 3 sites; total *n* = 180 fish) for seven days. Sentinel cages were constructed of mesh small enough to retain fry, but large enough to allow river water to flow through and provide food to the fish and actinospores to pass through unimpeded by the sentinel cage [54]. Cages were cabled to structures on the shore or to a dock in deep enough water so to remain submerged during all tide levels [54] but with slow enough water that fry could easily hold their position inside the cage, consistent with the type of habitat where chum salmon fry may be observed while migrating to the estuary [39,41]. Control groups of chum salmon fry from Big Creek hatchery (n = 15) and Washougal Hatchery (n = 15) were held in separate spore-free tanks at the lab concurrent with the sentinel exposure. These fish were held at 14°C and fed daily, and mortalities were recorded and examined for underlying cause [54].

Water samples were collected and processed as above, at sentinel cage sites on the first, fourth, and seventh days of the exposures to measure *C*. *shasta* densities and genotypes. At each sentinel site, *C*. *shasta* density was multiplied by the proportion of each genotype detected in the sample (i.e., genotype 0, I, II, or *C*. *gasterostea*) to obtain a daily mean density for each genotype. Mean exposure density was determined by averaging the daily genotype II densities from the three sample dates. Hourly temperatures (°C) were recorded at each sentinel site using a Pendant model Hobo temperature logger (Onset Computer Corp., Bourne, MA).

The remainder of the experiment and post-mortem evaluation occurred at the J.L. Fryer Aquatic Animal Health Laboratory (AAHL) at Oregon State University. Following field exposures, sentinel fish were placed in oxygenated coolers filled with river water and transported to the lab where they were held in separate 25 L tanks for 60 days post-exposure through 8 July on specific-pathogen-free (SPF) water. Water temperature in those tanks was 14°C (similar to the temperatures recorded at each sentinel site and within the optimum range for rearing juvenile chum salmon; [55]). Fish were fed daily and examined for clinical sings of disease, including reduced feeding and a darker coloration [27]. When clinical signs were observed, monitoring increased to twice daily. Mortalities were removed from the tank twice a day and necropsied. All remaining fish were euthanized on day 60 [54]. Fish were examined for *C*. *shasta* by collecting a swab from the hind gut post-mortem and examining a smear under a microscope (100-400x) for up to 3 minutes; swabs were retained for DNA extraction and sequencing. If *C*. *shasta* myxospores were not observed during the microscopy examination, a tissue sample was collected (1–3 cm piece of hindgut) for DNA extraction and PCR to confirm presence/ absence of *C*. *shasta* DNA (e.g., [56]). All positive samples were sequenced to determine genotype (0, I, and II; [19,20]).

To determine whether chum salmon were susceptible to lethal infection by *C*. *shasta* (i.e., that it was a mortality source for this ESA-listed species), it was necessary for death to be the endpoint of the study. If animals were euthanized when they began to show signs of disease, it would not be clear whether they could have otherwise eliminated the infection and survived. All experimental use and lethal take of chum salmon was reviewed and authorized under Animal Care and Use Permit 5010 and the Big Creek Hatchery Genetic Management Plan [57].

#### Analyses

We hypothesized that total % mortality and mean day to death (MDD) following the exposure to *C*. *shasta* would differ among sentinel sites in response to variation in *C*. *shasta* densities (i.e., dose) and that hatchery stocks derived from the Grays River and Lower Gorge populations would exhibit variable responses to *C*. *shasta* measured as total % mortality or MDD. We calculated total % mortality as the total number of *C*. *shasta*-related deaths (evidenced by visually detected spores or PCR positives) divided by the total number of fish exposed x 100. MDD was calculated as the number of days from the first day of exposure until *C*. *shasta*-related mortality occurred. We calculated adjusted total mortality by removing early mortalities from the pool. We compared the total % mortality among sentinel sites and between hatchery stocks using two separate chi-squared analyses [53]. Subsequently, we evaluated differences in MDD for the same fish among sentinel sites and between hatchery stocks. Because these data were not normally distributed, we used a non-parametric Kruskal Wallis test to evaluate differences and analyzed pairwise comparisons using the Wilcoxan rank sum test [53].

## Results

### Environmental conditions during study

Water temperature and discharge conditions preceding and concurrent with sample collection varied among years and between the Columbia and Willamette Rivers (Fig 1). During our sampling events, temperatures were lowest in 2018 and similar in 2019 and 2020 (Fig 1), but water temperatures were consistently higher in the Willamette River than in the Columbia River. In the Columbia River, the highest discharge occurred in 2018 (14,034 m^3^/s) in mid-May, overlapping with the last temporal sample event (Fig 1). In contrast, in 2018 peak discharge in the Willamette River was the lowest recorded during our three-year study period (2,562 m^3^/s). In 2019, a hundred-year magnitude flood (4,984 m^3^/s) occurred in the Willamette River in mid-April, immediately prior to our first sample event (Fig 1), whereas peak discharge that year in the Columbia River was the lowest recorded at that site during our study (10,118 m^3^/s). In 2020, moderate peak discharge occurred in early June in both rivers (Fig 1). Water temperature also varied among years and between the Columbia and Willamette Rivers (Fig 1). Water turbidity was influenced by both seasonal variation in the hydrograph and short-term rain events, and periods of increased turbidity negatively impacted the quality of water samples. In particular, a rain event in 2018 resulted in high turbidity during the fourth sample event. In 2019 and 2020, rain events did not produce turbidity, but it did increase at high tide for all tidally-influenced sites.

### Objective 1: Spatiotemporal distribution of *C*. *shasta*

*Ceratonova shasta* was detected at 23 of 29 (79.3%) mainstem Columbia River sites (Fig 2; Table 3) and at 19 of 29 (65.5%) tributary sites (Fig 2; Table 4), 2018–2020. Genotype I was detected at 7 sites on the Columbia River mainstem and in 13 tributaries, whereas genotype II was detected at 21 sites on the Columbia River mainstem and in 16 tributaries (Fig 2; Tables 3 and 4). In three locations (downstream Lewis and Clark River site and two sites in the Columbia River, *C*. *shasta* was detected but could not be sequenced because of inhibition (Fig 2; Tables 3 and 4). Genotype 0 was not detected in any samples but *C*. *gasterostea* was detected at 12 sites on the Columbia River and in 9 tributaries (Fig 2; Tables 3 and 4). In 2018, high inhibition may have obscured potential positive detections at low *C*. *shasta* densities (< 2 spores/ L), but this was addressed in 2019 and 2020 by processing smaller volumes of water per filter, and many low-level detections (< 2 spore/ L) were observed Although variation in spore density was present among sites, very little variation in spore density was observed among individual liters within a site for a given sample date. In 2019, the average CV was 0.55 (range = 0.05–1.18) and in 2020, the average CV was 0.88 (range = 0.04–1.73).

**Table 3:** Sample site code, *Ceratonova shasta* density (spores/ L), and genotypes (subscript 1 = I, 2 = II, u = unknown) measured at Columbia River sites 2018–2020. The table excludes the first two sample events in 2018 as no *C*. *shasta* was detected. The table also excludes sample sites where *C*. *shasta* was never detected. Empty cells indicate the site was not sampled during a particular sample event.

Site code		2018			2019				2020		
(State)	4/2	4/17	5/1	4/15	5/1	5/15	4/15	4/22	5/1	5/7	5/15
1 (OR)	0	< 2_1,2_	0	0	0	2.47_1,2_					< 2_1,2_*
2 (WA)	0	< 2_1,2_	0	0	0	< 2_1_					< 2_2_
4 (OR)							0	0	< 2_2_	2.45_2_	< 2_2_
5 (OR)										< 2_2_	
6 (OR)			0		2.15_2_		< 2_1,2_	< 2_2_	2.13_1,2_	< 2_2_	5.57_1,2_
9 (OR)										< 2_2_	
10 (OR)										< 2_1,2_*	
11 (OR)								< 2_2_	< 2_u_	< 2_u_	< 2_2_
12 (OR)										< 2_u_	
13 (WA)										< 2_2_	
14 (OR)										< 2_2_	
15 (WA)										< 2_2_	
18 (OR)							< 2_2_	0*	< 2_2_	< 2_u_	0*
19 (OR)			0		2.26_2_		< 2_2_	< 2_2_	2.23_2_	< 2_2_	< 2_2_*
21 (OR)										< 2_2_	
22 (OR)					3.69_2_						
23 (OR)	0	2.5_u_			< 2_2_	< 2_2_	6.27_2_	< 2_2_	9.7_2_	2.21_2_*	7.93_1,2_
24 (OR)			2.28_1,2_		< 2_2_*						
25 (OR)			< 2_2_*		0						
26 (OR)			< 2_1,2_		< 2_1_						
27 (OR)			< 2_u_		< 2_u_*						
28 (OR)					< 2_2_*					< 2_2_*	
29 (OR)					< 2_2_*		0	< 2_u_			< 2_u_

*C. *gasterostea* detected but not reported in spore total.

**Table 4:** Sample site code, *Ceratonova shasta* density (spores/ L), and genotypes (subscript 1 = I, 2 = II, u = unknown) measured at tributary sites in the lower Columbia River Basin, 2018–2020. INH indicates the qPCR reaction was inhibited and spores could not be quantified or genotyped. The table excludes sample sites where *C*. *shasta* was never detected. Blank cells indicate the site was not sampled during a particular sample event.

Site code (State)		2018					2019				2020		
	3/5	3/19	4/2	4/17	5/1	4/15	5/1	5/15	4/15	4/22	5/1	5/7	5/15
31 (OR)	0	INH	0	0	6.61_u_	0	2.69_1_	3.57_1,2_					
32 (OR)									< 2_2_	< 2_2_	2.83_1,2_	6.37_2_	8.37_2_
33 (OR)	0	INH	28.45_1,2_	< 2_u_	34.63_2_	0	< 2_1,2_	5.24_2_	< 2_1_	< 2_2_	2.93_2_	5.77_1,2_	7.4_2_
34 (OR)									< 2_1,2_	< 2_2_	7.32_1,2_*	4.6_1,2_	12.1_1,2_
35 (OR)					0		< 2_1,2_						
36 (OR)	0	INH	< 2_1,2_	0	0	0	0	0					
37 (OR)	0	INH	0	< 2_u_	0	2_2_	5.33_2_	< 2_2_	X	2.67_2_		0.87_2_	0.2_2_
38 (OR)	0	INH	0	0	3.82_2_	0	< 2_2_*	< 2_2_	0	0*	< 2_u_	0*	0.38_1_*
39 (OR)					0		< 2_2_*						0
42 (OR)							< 2_1,2_						
43 (OR)					INH		< 2_2_*						
44 (OR)					3.77_1,2_		3.05_2_						
46 (OR)	7.72_u_					0	0	0					
47 (OR)		INH	0	0	2.75_1,2_	0	< 2_2_*	0*	0	0	0	0	0.03_u_
48 (WA)	0	INH	0	0	0*	0	0*	0					0.23_2_
51 (WA)	0	INH	0	0	0	0	2.39_1,2_	5.22_2_*					
53 (WA)	0	INH	0	0	< 2_1_	0	0	0					0
55 (WA)	0	2.06_1_	17.09_1_	3.58_u_	79.49_1_	0	10.53_1_	13.45_1_					5.07_1_
57 (WA)	0	INH	0	0	0	0	0	3.05_2_				0.03_u_	0.03_u_

*C. *gasterostea* detected but not reported in spore total.

Presence and density of *C*. *shasta* varied among Columbia River and tributary sites seasonally and among years (Tables 3 and 4). In general, *C*. *shasta* was first detected in mid to late April in temporal samples (Tables 3 and 4). The earliest detection of *C*. *shasta* occurred at a single tributary site (Lewis and Clark River) on 5 March, 2018 (Tables 3 and 4). The earliest *C*. *shasta* detection in the Columbia River occurred on the April 15^th^ sample date each year and it was detected in over 70% of sampled sites by May 1. At sites where both genotype I and II were detected, genotype I was generally detected earlier than genotype II (Tables 3 and 4). Overall, densities of *C*. *shasta* were highest in tributary sites in 2018 (range = 2.06–79.5 spores/L) and lower but similar in 2019 (range = 2–13.45 spores/L) and 2020 (range = < 2–12.1 spores/ L). At mainstem sites densities were highest in 2020 (range = < 2–9.7 spores/L) and lower but similar in 2018 (range = < 2–2.5 spores/L) and 2019 (range = < 2–3.69 spores/L; Tables 3 and 4).

In tributaries, *C*. *shasta* genotype II was detected in 3/5 historical spawning streams and 10/13 intermittent spawning streams (Tables 4 and 5). It was not detected at any contemporary spawning streams during the period in which chum salmon fry inhabit or outmigrate from those streams, however it was detected in two Washington streams after May 1 (Tables 4 and 5), > 2 weeks after chum salmon fry had emigrated from the streams. This negative relationship between the presence of *C*. *shasta* and the absence of contemporary spawning by chum salmon was highly significant (χ^2^ = 10.73, df = 1, *p* = 0.001; Table 5).

**Table 5:** Site code (state), chum salmon *Oncorhynchus keta* spawning status (historically present, intermittently present, or consistently present), and presence of *Ceratonova shasta* genotype II in tributaries and the closest Columbia River sample sites during the season when juveniles are present (Mar–May 1 in tributaries, Mar–May in the Columbia River), 2018–2020.

Tributary site code (state)	Chum status	*C*. *shasta* (II) detected in tributary Mar—May 1	Closest Columbia River sample site code (state)	*C*. *shasta* (II) detected in Columbia River Mar–May
30 (OR)	Historical	No	4 (OR)	Yes
31 (OR)	Intermittent	Yes	6 (OR)	Yes
32 (OR)	Intermittent	Yes	6 (OR)	Yes
33 (OR)	Intermittent	Yes	6 (OR)	Yes
34 (OR)	Intermittent	Yes	6 (OR)	Yes
35 (OR)	Historical	Yes	11 (OR)	Yes
36 (OR)	Historical	Yes	18 (OR)	Yes
37 (OR)	Historical	Yes	18 (OR)	Yes
38 (OR)	Intermittent	Yes	19 (OR)	Yes
39 (OR)	Intermittent	Yes	19 (OR)	Yes
40 (OR)	Present	No	24 (OR)	Yes
41 (OR)	Present	No	26 (OR)	Yes
42 (OR)	Intermittent	Yes	28 (OR)	Yes
43 (OR)	Intermittent	Yes	29 (OR)	Yes
44 (OR)	Intermittent	Yes	29 (OR)	Yes
45 (OR)	Historical	No	29 (OR)	Yes
46 (OR)	Intermittent	Yes	NA	NA
47 (OR)	Intermittent	Yes	NA	NA
48 (WA)	Present	No	2 (WA)	Yes
49 (WA)	Present	No	3 (WA)	No
50 (WA)	Present	No	3 (WA)	No
50 (WA)	Intermittent	Yes	4 (OR)	Yes
51 (WA)	Present	No	10 (OR)	Yes
52 (WA)	Present	No	10 (OR)	Yes
53 (WA)	Intermittent	No	13 (WA)	Yes
54 (WA)	Present	No	15 (WA)	Yes
55 (WA)	Present	No	20 (WA)	No
56 (WA)	Present	No	NA	NA
57 (WA)	Present	No	NA	NA

### Objective 2: Sentinel study

In general, water temperature and *C*. *shasta* densities increased over the exposure period May 1 –May 8 at all sites. During the sentinel exposure, *C*. *shasta* genotype II was detected at all sites, genotype I was detected at the Willamette River site only, and *C*. *gasterostea* was detected at the Tongue Point and Lewis and Clark River sites only. The densities of genotype II detected during the sentinel exposures were lower than those measured in 2018; during the exposure the highest densities were measured at the Willamette River site (mean = 2 spores/ L; Table 6). At both Tongue Point and the Lewis and Clark River, the *C*. *shasta* densities were < 2 spores/ L (Table 6). Water temperature ranged from 12.9–17.3°C across sites (Table 6). Although our measurements of spore density were grab samples and were not adjusted for differences in discharge among the sites, they reflect the variation in *C*. *shasta* densities among sites. A total of four fry died during the sentinel exposure (2 in cages and 2 during transport to the AAHL). These mortalities were subtracted from the totals observed during rearing at AAHL and were not attributed to *C*. *shasta*.

**Table 6:** Average density (spores/ L) and range of *Ceratonova shasta* genotype II from three water samples, genotypes present (0, I, II, or *C*. *gasterostea*), and range of mean daily temperatures (°C) at sentinel sites on the Willamette River, Tongue Point (Columbia River) and the Lewis and Clark River in the Columbia Basin, May 1- May 8, 2019.

Site	Avg spores/ L genotype II (range)	Genotypes present	Range of mean daily temperature (°C)
Willamette R.	2 (< 2–3.05)	I and II	12.9–16.5
Columbia R. at Tongue Point	< 2	II, *C*. *gasterostea*	13.6–14.8
Lewis and Clark R.	< 2	II, *C*. *gasterostea*	14.9–17.3

**Fig 3:**
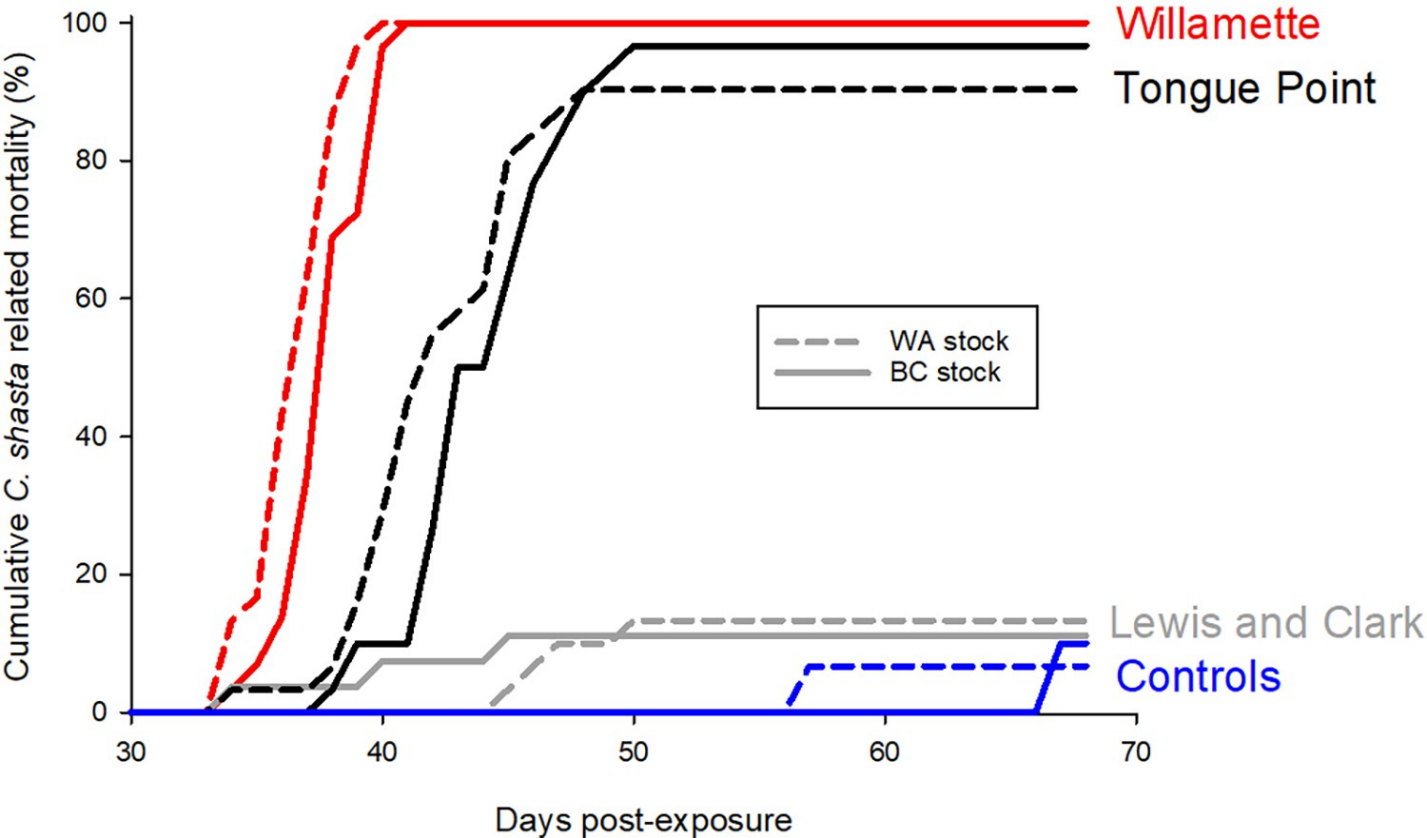
Cumulative percent mortality of chum salmon *Oncorhynchus keta* fry from Washougal Hatchery (WA stock) and Big Creek Hatchery (BC stock) exposed to *Ceratonova shasta* at sentinel sites on the Willamette River, Columbia River at Tongue Point, and Lewis and Clark River. Mortalities are only included if *C*. *shasta* myxospores were observed or if infection was confirmed through PCR. Mortality in hatchery control groups was from natural causes (n = 1).

Mortality attributed to *C*. *shasta* differed among sites (χ^2^ = 130.41, df = 2, *p* < 0.001) but not between hatchery stocks (χ^2^ = 0.06, df = 1, *p* = 0.81). At the Willamette River site, 100% of Washougal Hatchery fish died (n = 30/ 30) and 100% of Big Creek Hatchery fish died (n = 29/ 29; Fig 2). All mortalities were positive for *C*. *shasta* through either observation of myxospores (n = 58) or detection of *C*. *shasta* DNA by PCR (n = 1). At the Columbia River site at Tongue Point, 96.7% of Washougal fish (n = 30/ 31) and 100% of Big Creek fish (n = 30/ 30) died (Fig 3). Adjusted *C*. *shasta* mortality was 90.3% for Washougal fish (n = 28/ 31) and 96.6% for Big Creek fish (n = 29/ 30); 49 fish were positive by visual examination and 8 were positive by PCR. At the Lewis and Clark River site, 30% of Washougal fish died (n = 9/ 31) and 66% of Big Creek fish died (n = 18/ 27; Fig 2). Adjusted *C*. *shasta* mortality was 13.3% for Washougal fish (n = 4/ 30) and 11.1% for Big Creek fish (n = 3/ 27); 5 fish were positive by visual examination and 2 were positive by PCR. In the remaining fish, mortality was not attributed to *C*. *shasta*.

Total days until death differed significantly among sentinel sites (Kruskal-Wallis χ^2^ = 70.329, df = 2, *p* < 0.001). Mortality occurred from days 34 to 41 in fish exposed at the Willamette River site, from days 34 to 50 at Tongue Point on the Columbia River, and from days 34 to 50 at the Lewis and Clark River. River. Mortality occurred significantly earlier in the Willamette River (mean = 37.4) when compared to either Tongue Point (mean = 43.1) or the Lewis and Clark River (mean = 43.9; Wilcoxan Rank Sum Test *p* < 0.001 and *p* = 0.005, respectively; Fig 3); Tongue Point and the Lewis and Clark River were not significantly different from each other (Wilcoxan Rank Sum Test *p* = 0.34). Across all sites, fry from Washougal Hatchery died slightly faster than fry from Big Creek Hatchery, however total days until death did not differ significantly between hatchery stocks at *α* = 0.05 (Kruskal-Wallis χ^2^ = 3.2062, df = 1, *p* = 0.07).

## Discussion

Recovery efforts for threatened and endangered salmon and steelhead in the CRB primarily focus on mitigating mortality driven by the 4 Hs (Hatcheries, Harvest, Hydrosystem, and Habitat). However, parasites and disease also contribute to mortality and have potential to hamper recovery efforts. In this study, we described the spatial and temporal distribution of the myxozoan salmonid parasite *C*. *shasta* within tributaries and the Lower Columbia River mainstem to assess potential overlap with habitats occupied currently or historically by juvenile chum salmon. We assessed prevalence of infection and mortality in juvenile chum salmon exposed to a range of ambient *C*. *shasta* densities at tributary and Columbia River sites. We interpret these data below in the context of chum salmon recovery efforts in the CRB. These data represent the first investigation of the effects of *C*. *shasta* on ESA-listed Columbia River chum salmon and indicate its potential to be a limiting factor.

*Ceratonova shasta* genotype II was distributed throughout tributaries in which chum salmon were extirpated or only intermittently present and was not detected in contemporary spawning tributaries during the timeframe juvenile chum salmon are present. These positive tributary detections occurred primarily in Oregon; of 18 Oregon tributaries sampled, genotype II was found in 14. Genotype II was also detected throughout the Columbia River mainstem in at least 21 of 29 sites in Oregon and Washington; these detections occurred in locations where chum salmon rear or migrate. High densities of genotype II were detected in Beaver and Knappa Sloughs in Oregon. These detections were significant because Beaver Slough drains tributaries where (unsuccessful) chum salmon reintroductions have occurred [51], and Knappa Slough drains tributaries with extant populations, including a release-site for the chum salmon conservation broodstock. In addition, Knappa Slough is rearing habitat for juvenile chum salmon from throughout the CRB [38,58]. Although the focus of this study was understanding the distribution of genotype II because of infection risk to chum salmon, describing the distribution of genotype I is also critical to understanding risk. Wherever genotype I is detected, we know the invertebrate host is present, so there is potential for genotype II to appear in those streams if myxospores are introduced by a salmonid host.

The timing of *C*. *shasta* detection varied among years but overlapped partially with the timing of juvenile chum salmon outmigration from tributaries and substantially with juvenile rearing in the lower Columbia River. Among study years *C*. *shasta* was detected earliest at sites where chum salmon historically spawned (now extirpated) or are only present intermittently. At these sites, *C*. *shasta* was detected as early as March 5^th^ and was consistently detected by April 15th. This period overlaps with the period when juvenile chum salmon migrate from their natal streams [47,48]. In contrast, detections in two contemporary spawning tributaries both occurred after May 1, when juvenile chum salmon were no longer present. At Columbia River mainstem sites, *C*. *shasta* was typically detected by April 15^th^, which overlapped with the period when juvenile chum salmon are present demonstrating clear potential for infection and disease risk. Variation in the timing when *C*. *shasta* was first detected each year further suggests that its significance as a mortality factor may vary annually.

Variation in timing and density of *C*. *shasta* likely corresponded with broad-scale differences in water temperature and discharge. In the Willamette River, *C*. *shasta* densities were substantially lower in 2019 than those measured in 2018. We suggest the lower densities in 2019 are explained by the large (100-year magnitude) flood that occurred immediately prior to water sampling and sentinel exposures in 2019. In addition, water temperatures were highest in 2018, driving the relatively high *C*. *shasta* densities measured that year. Variation in spore density has been described on the Klamath River in response to variations in discharge and temperature [11,25]. When discharge is high enough to scour the substrate, it can displace the worm host and lead to lower spore densities [8,59]. Conversely, higher temperatures can result in faster completion of the parasite life cycle [60] and higher parasite replication rates [16], leading to earlier detection and higher densities. Further research is needed on the relationship between temporal variation in *C*. *shasta* and environmental and biological conditions in the Columbia River and tributaries.

Variation in spore density also occurred for reasons other than seasonal or annual differences in environmental conditions. At tidally-influenced sites (still freshwater) such as Knappa Slough, Multnomah Channel, or the Willamette River at Willamette Park, spore density varied substantially among weeks. This variation was likely related to the diel timing of sample collection relative to the tidal cycle (low or high tide); sample size was insufficient to statistically evaluate that pattern. Regardless, this variation does corroborate other observations that *C*. *shasta* density varies spatially and temporally and suggests that an incoming tide could temporarily alter local spore densities. Variation in spore density was also observed among the individual liters of water collected for a single site.

Juvenile chum salmon from Big Creek and Washougal Hatcheries experienced substantial mortality at low spore densities across sentinel sites, demonstrating that they are highly susceptible to lethal infection from this parasite. All chum salmon at the Willamette River site died following exposure to 2 spores/L. In the Lewis and Clark and Columbia Rivers, lethal infections occurred at densities < 2 spores/ L, but mortality rates differed between sites. We measured similar densities of *C*. *shasta* at both sites but the greater discharge in the Columbia River (relative to the Lewis and Clark River) resulted in a greater total number of spores encountered in the same amount of time explaining the higher mortality in fish exposed there. This point can be illustrated by comparing estimated spore exposure between the Willamette and Columbia Rivers- the two sites with available discharge data. If we assume all *C*. *shasta* detections were from actinospores and expand the measured *C*. *shasta* densities by the discharge at those two sites during the sentinel study (*sensu* [11]), we estimate *daily* spore densities of 9.8 X 10^10^ and 2.53 X 10^11^, respectively. Therefore, even at spore densities at or below the detection threshold, fish are theoretically exposed to a tremendous quantity of spores over the course of a day. In other Columbia River species, mortality typically occurs at much higher *C*. *shasta* densities than what was observed for chum salmon in this study [7]. For example, at ambient spore densities, *infection* rates were only 5–12% for coho salmon, Chinook salmon, and steelhead [7] in the CRB. Additional research is needed across a range of low spore densities to determine the infectious threshold for juvenile chum salmon. This would allow further exploration of the specific time frame when *C*. *shasta* densities are high enough in tributaries to cause lethal infection.

The time from infection to death for juvenile chum salmon ranged from 34 to 50 days, across ambient spore densities. This time frame was similar to observations in Coho Salmon and other species infected with genotype II [11]. For chum salmon, the progression from infection to death suggests they would succumb to infection either during estuary residency or shortly after ocean entrance, depending on where exposure occurred, the exposure dose (spore density), water temperature, and the presence of other stressors. Juvenile chum salmon infected with *C*. *shasta* have been captured in the ocean [42], indicating that smolting or entering saltwater do not eliminate the infection. However, the rate of progression from infection to disease in saltwater is not known.

## Conclusions

In this study, we demonstrated that *C*. *shasta* genotype II causes mortality in juvenile chum salmon at ambient spore densities and that it overlaps spatially with tributary spawning habitat from which chum salmon have been extirpated or are only present intermittently. We further observed that a current portion of outmigrating chum salmon overlap temporally with *C*. *shasta*, indicating some level of mortality is likely each year. Although *C*. *shasta* was known to occur in the CRB [7,17,29,60], this study provided the first fine-scale assessment of distribution downstream of Bonneville Dam, expanding known detections of this parasite in relation to the historical and contemporary chum salmon habitat. The detections of genotype II in the Grays River and Hamilton Creek (both after May 1st) were potentially concerning. Both sites are population strongholds and are critical to the persistence of the Columbia River ESU [61–63]. Following warmer water temperatures and decreased river flows, *C*. *shasta* may be present in the water column earlier in the year, overlap with a greater portion of outmigrating fry, and occur at higher densities [64]. Any expansion of *C*. *shasta* distribution earlier in the year or into additional tributaries could complicate efforts to recover chum salmon, particularly in Oregon, where few tributaries were found that did not contain *C*. *shasta* genotype II. As such, additional research is needed to characterize the degree to which *C*. *shasta* limits the survival or distribution of chum salmon currently and under future climate scenarios.

## Supporting information

S1 Table Sample site code, *Ceratonova shasta* density (spores/ L), and genotypes (subscript 1 = I, 2 = II, u = unknown) measured at all Columbia River sites 2018–2020. INH indicates the qPCR reaction was inhibited and spores could not be quantified or genotyped. “X” indicates the site was not sampled during a particular sample event. *C. *gasterostea* detected but not reported in spore total.
(DOCX)

S2 Table Sample site code, *Ceratonova shasta* density (spores/ L), and genotypes (subscript 1 = I, 2 = II, u = unknown) measured at all tributary sites in the lower Columbia River Basin, 2018–2020. INH indicates the qPCR reaction was inhibited and spores could not be quantified or genotyped. “X” indicates the site was not sampled during a particular sample event. *C. *gasterostea* detected but not reported in spore total.
(DOCX)
