# Supplementary material for: Spatiotemporal distribution of Ceratonova shasta in the lower Columbia River Basin and effects of exposure on survival of juvenile chum salmon Oncorhynchus keta
Source: PLoS One. 2022 Aug 26;17(8):e0273438. doi: 10.1371/journal.pone.0273438 (PMC9417023; doi:10.1371/journal.pone.0273438)
Supplement: S1 Table — INH indicates the qPCR reaction was inhibited and spores could not be quantified or genotyped. “X” indicates the site was not sampled during a particular sample event. *C. gasterostea detected but not reported in spore total. (DOCX) [file pone.0273438.s001.docx]

S1 Table. Sample site code, *Ceratonova shasta* density (spores/ L), and genotypes (subscript 1=I, 2 = II, u =unknown) measured at all Columbia River sites 2018-2020. INH indicates the qPCR reaction was inhibited and spores could not be quantified or genotyped. “X” indicates the site was not sampled during a particular sample event. **C. gasterostea* detected but not reported in spore total.

| Site code |  |  | | 2018 |  | |  |  | 2019 |  |  |  | | 2020 |  | |  |
| --- | --- | --- | --- | --- | --- | --- | --- | --- | --- | --- | --- | --- | --- | --- | --- | --- | --- |
| (State) | 3/5 | | 3/19 | 4/2 | 4/17 | 5/1 | | 4/15 | 5/1 | 5/15 | 4/15 | | 4/22 | 5/1 | 5/7 | 5/15 | |
| 1 (OR) | 0 | | INH | 0 | < 2_1,2_ | 0 | | 0 | 0 | 2.47_1,2_ | X | | X | X | X | < 2_1,2_* | |
| 2 (WA) | 0 | | INH | 0 | < 2_1,2_ | 0 | | 0 | 0 | < 2_1_ | X | | X | X | X | < 2_2_ | |
| 3 (WA) | X | | X | X | X | 0 | | X | 0 | X | X | | X | X | X | X | |
| 4 (OR) | X | | X | X | X | X | | X | X | X | 0 | | 0 | < 2_2_ | 2.45_2_ | < 2_2_ | |
| 5 (OR) | X | | X | X | X | X | | X | X | X | X | | X | X | < 2_2_ | X | |
| 6 (OR) | X | | X | X | X | 0 | | X | 2.15_2_ | X | < 2_1,2_ | | < 2_2_ | 2.13_1,2_ | < 2_2_ | 5.57_1,2_ | |
| 7 (OR) | X | | X | X | X | X | | X | X | X | X | | X | X | 0* | X | |
| 8 (OR) | X | | X | X | X | X | | X | X | X | X | | X | X | 0 | X | |
| 9 (OR) | X | | X | X | X | X | | X | X | X | X | | X | X | < 2_2_ | X | |
| 10 (OR) | X | | X | X | X | X | | X | X | X | X | | X | X | < 2_1,2_* | X | |
| 11 (OR) | X | | X | X | X | X | | X | X | X | X | | < 2_2_ | < 2_u_ | < 2_u_ | < 2_2_ | |
| 12 (OR) | X | | X | X | X | X | | X | X | X | X | | X | X | < 2_u_ | X | |
| 13 (WA) | X | | X | X | X | X | | X | X | X | X | | X | X | < 2_2_ | X | |
| 14 (OR) | X | | X | X | X | X | | X | X | X | X | | X | X | < 2_2_ | X | |
| 15 (WA) | X | | X | X | X | X | | X | X | X | X | | X | X | < 2_2_ | X | |
| 16 (OR) | X | | X | X | X | X | | 0 | X | X | X | | X | X | X | X | |
| 17 (OR) | X | | X | X | X | X | | 0 | X | X | X | | X | X | X | X | |
| 18 (OR) | X | | X | X | X | X | | X | X | X | < 2_2_ | | 0* | < 2_2_ | < 2_u_ | 0* | |
| 19 (OR) | X | | X | X | X | 0 | | X | 2.26_2_ | X | < 2_2_ | | < 2_2_ | 2.23_2_ | < 2_2_ | < 2_2_* | |
| 20 (WA) | X | | X | X | X | X | | X | X | X | X | | X | X | 0* | X | |
| 21 (OR) | X | | X | X | X | X | | X | X | X | X | | X | X | < 2_2_ | X | |

S1 Table. (continued)

| Site code |  |  | | 2018 |  | |  |  | 2019 |  |  |  | | 2020 |  | |  |
| --- | --- | --- | --- | --- | --- | --- | --- | --- | --- | --- | --- | --- | --- | --- | --- | --- | --- |
| (State) | 3/5 | | 3/19 | 4/2 | 4/17 | 5/1 | | 4/15 | 5/1 | 5/15 | 4/15 | | 4/22 | 5/1 | 5/7 | 5/15 | |
| 22 (OR) | X | | X | X | X | X | | X | 3.69_2_ | X | X | | X | X | X | X | |
| 23 (OR) | 0 | | INH | 0 | 2.5_u_ | X | | X | < 2_2_ | < 2_2_ | 6.27_2_ | | < 2_2_ | 9.7_2_ | 2.21_2_* | 7.93_1,2_ | |
| 24 (OR) | X | | X | X | X | 2.28_1,2_ | | X | < 2_2_* | X | X | | X | X | X | X | |
| 25 (OR) | X | | X | X | X | < 2_2_* | | X | 0 | X | X | | X | X | X | X | |
| 26 (OR) | X | | X | X | X | < 2_1,2_ | | X | < 2_1_ | X | X | | X | X | X | X | |
| 27 (OR) | X | | X | X | X | < 2_u_ | | X | < 2_u_* | X | X | | X | X | X | X | |
| 28 (OR) | X | | X | X | X | X | | X | < 2_2_* | X | X | | X | X | < 2_2_* | X | |
| 29 (OR) | X | | X | X | X | X | | X | < 2_2_* | X | 0 | | < 2_u_ | X | X | < 2_u_ | |
