# Supplementary material for: Spatiotemporal distribution of Ceratonova shasta in the lower Columbia River Basin and effects of exposure on survival of juvenile chum salmon Oncorhynchus keta
Source: PLoS One. 2022 Aug 26;17(8):e0273438. doi: 10.1371/journal.pone.0273438 (PMC9417023; doi:10.1371/journal.pone.0273438)
Supplement: S2 Table — INH indicates the qPCR reaction was inhibited and spores could not be quantified or genotyped. “X” indicates the site was not sampled during a particular sample event. *C. gasterostea detected but not reported in spore total. (DOCX) [file pone.0273438.s002.docx]

S2 Table. Sample site code, *Ceratonova shasta* density (spores/ L), and genotypes (subscript 1=I, 2 = II, u =unknown) measured at all tributary sites in the lower Columbia River Basin, 2018-2020. INH indicates the qPCR reaction was inhibited and spores could not be quantified or genotyped. “X” indicates the site was not sampled during a particular sample event. **C. gasterostea* detected but not reported in spore total.

| Site code (State) |  | 2018 | | | | |  | | 2019 | | |  |  | | 2020 |  |  | |
| --- | --- | --- | --- | --- | --- | --- | --- | --- | --- | --- | --- | --- | --- | --- | --- | --- | --- | --- |
|  | 3/5 | | 3/19 | 4/2 | 4/17 | 5/1 | | 4/15 | | 5/1 | 5/15 | 4/15 | 4/22 | | 5/1 | 5/7 | 5/15 | |
| 30 (OR) | 0 | | INH | 0 | 0 | 0 | | 0 | | 0 | 0 | X | X | X | | X | | X |
| 31 (OR) | 0 | | INH | 0 | 0 | 6.61_u_ | | 0 | | 2.69_1_ | 3.57_1,2_ | X | X | X | | X | | X |
| 32 (OR) | X | | X | X | X | X | | X | | X | X | < 2_2_ | < 2_2_ | 2.83_1,2_ | | 6.37_2_ | | 8.37_2_ |
| 33 (OR) | 0 | | INH | 28.45_1,2_ | < 2_u_ | 34.63_2_ | | 0 | | < 2_1,2_ | 5.24_2_ | < 2_1_ | < 2_2_ | 2.93_2_ | | 5.77_1,2_ | | 7.4_2_ |
| 34 (OR) | X | | X | X | X | X | | X | | X | X | < 2_1,2_ | < 2_2_ | 7.32_1,2_* | | 4.6_1,2_ | | 12.1_1,2_ |
| 35 (OR) | X | | X | X | X | 0 | | X | | < 2_1,2_ | X | X | X | X | | X | | X |
| 36 (OR) | 0 | | INH | < 2_1,2_ | 0 | 0 | | 0 | | 0 | 0 | X | X | X | | X | | X |
| 37 (OR) | 0 | | INH | 0 | < 2_u_ | 0 | | 2_2_ | | 5.33_2_ | < 2_2_ | X | 2.67_2_ | X | | 0.87_2_ | | 0.2_2_ |
| 38 (OR) | 0 | | INH | 0 | 0 | 3.82_2_ | | 0 | | < 2_2_* | < 2_2_ | 0 | 0* | < 2_u_ | | 0* | | 0.38_1_* |
| 39 (OR) | X | | X | X | X | 0 | | X | | < 2_2_* | X | X | X | X | | X | | 0 |
| 40 (OR) | 0 | | INH | 0 | 0 | 0 | | 0 | | 0 | 0 | X | X | X | | X | | 0 |
| 41 (OR) | 0 | | INH | 0 | 0 | 0 | | 0 | | 0 | 0 | X | X | X | | X | | X |
| 42 (OR) | X | | X | X | X | X | | X | | < 2_1,2_ | X | X | X | X | | X | | X |
| 43 (OR) | X | | X | X | X | INH | | X | | < 2_2_* | X | X | X | X | | X | | X |
| 44 (OR) | X | | X | X | X | 3.77_1,2_ | | X | | 3.05_2_ | X | X | X | X | | X | | X |
| 45 (OR) | X | | X | X | X | 0 | | X | | X | X | X | X | X | | X | | X |
| 46 (OR) | 7.72_u_ | | X | X | X | X | | 0 | | 0 | 0 | X | X | X | | X | | X |
| 47 (OR) | X | | INH | 0 | 0 | 2.75_1,2_ | | 0 | | < 2_2_* | 0* | 0 | 0 | 0 | | 0 | | 0.03_u_ |

S2 Table. (continued)

| Site code (State) |  |  | | 2018 |  | |  | |  | 2019 |  |  | |  | | 2020 |  | |  |
| --- | --- | --- | --- | --- | --- | --- | --- | --- | --- | --- | --- | --- | --- | --- | --- | --- | --- | --- | --- |
|  | 3/5 | | 3/19 | 4/2 | 4/17 | 5/1 | | 4/15 | | 5/1 | 5/15 | | 4/15 | | 4/22 | 5/1 | 5/7 | 5/15 | |
| 48 (WA) | 0 | | INH | 0 | 0 | 0* | | 0 | | 0* | 0 | | X | | X | X | X | 0.23_2_ | |
| 49 (WA) | X | | X | X | X | X | | X | | 0* | X | | X | | X | X | X | 0 | |
| 50 (WA) | X | | X | X | X | X | | X | | 0 | X | | X | | X | X | X | 0 | |
| 50 (WA) | X | | X | X | X | X | | X | | 0* | X | | X | | X | X | X | 0 | |
| 51 (WA) | 0 | | INH | 0 | 0 | 0 | | 0 | | 2.39_1,2_ | 5.22_2_* | | X | | X | X | X | X | |
| 52 (WA) | X | | X | X | X | 0* | | X | | 0* | X | | X | | X | X | X | X | |
| 53 (WA) | 0 | | INH | 0 | 0 | < 2_1_ | | 0 | | 0 | 0 | | X | | X | X | X | 0 | |
| 54 (WA) | X | | X | X | X | 0 | | X | | 0 | X | | X | | X | X | X | 0 | |
| 55 (WA) | 0 | | 2.06_1_ | 17.09_1_ | 3.58_u_ | 79.49_1_ | | 0 | | 10.53_1_ | 13.45_1_ | | X | | X | X | X | 5.07_1_ | |
| 56 (WA) | X | | X | X | X | 0 | | X | | 0 | X | | X | | X | X | X | X | |
| 57 (WA) | 0 | | INH | 0 | 0 | 0 | | 0 | | 0 | 3.05_2_ | | X | | X | X | 0.03_u_ | 0.03_u_ | |
| 58 (WA) | X | | X | X | X | X | | 0 | | 0 | 0 | | X | | X | X | 0 | 0 | |
